# 887. Burn Care in Prisons: Ethical Dilemmas in Correctional Health Care

**DOI:** 10.1093/jbcr/irag033.373

**Published:** 2026-04-06

**Authors:** Joshua Khorsandi, Liahm Blank, Samir Alkhouri, Abu-Bakr Ahmed, Kathryn Cataldo

**Affiliations:** Kirk Kerkorian School of Medicine at University of Nevada, Las Vegas, NV; Kirk Kerkorian School of Medicine at University of Nevada, Las Vegas, NV; Kirk Kerkorian School of Medicine at University of Nevada, Las Vegas, NV; Kirk Kerkorian School of Medicine at University of Nevada, Las Vegas, NV; Department of Plastic and Reconstructive Surgery, University of Nevada, Las Vegas, NV

## Abstract

**Introduction:**

Burn injuries among incarcerated individuals present an underexplored intersection of medical ethics, correctional policy, and health equity. While the literature increasingly acknowledges the vulnerabilities of populations such as refugees and undocumented patients, incarcerated burn patients remain largely absent from scholarly and professional discussions. The principle of dual loyalty, in which clinicians must balance the duty of care to the patient with obligations to the institution or state, creates unique challenges in correctional settings. These challenges are magnified in burn care, where timely interventions, pain management, rehabilitation, and psychosocial support are critical to recovery but often constrained by security protocols and systemic neglect.

**Methods:**

A narrative review was conducted across PubMed, Embase, and Scopus to identify peer-reviewed articles, position statements, and ethical analyses addressing medical care for incarcerated populations, dual loyalty, and burn injury treatment. Inclusion criteria emphasized works published in English from 2000 to 2025. Thematic synthesis was employed to identify recurring ethical tensions, clinical barriers, and potential frameworks for resolution.

**Results:**

The review revealed a significant gap: while extensive ethical discourse exists on the care of vulnerable groups such as undocumented migrants or patients with limited access to healthcare, incarcerated burn patients are rarely addressed. Key themes included compromised access to specialized burn centers, ethical dilemmas surrounding informed consent under custodial oversight, inadequate rehabilitative care, and disproportionate suffering due to institutional neglect. The dual loyalty conflict emerged as central, often leaving clinicians torn between patient dignity and correctional authority demands.

**Conclusions:**

The invisibility of incarcerated burn patients in both the literature and professional discourse represents a neglected ethical frontier. Addressing these gaps requires elevating the issue within burn care scholarship and advocacy platforms.

**Applicability to Practice:**

For burn surgeons and allied professionals, recognizing the ethical complexities of treating incarcerated patients is essential to uphold standards of care. By explicitly addressing dual loyalty, practitioners can advocate for systemic reforms that preserve patient dignity while maintaining institutional safety. This discourse has the potential to reshape policy, improve clinical outcomes, and reaffirm the profession’s ethical commitments.

